# Tendencies in the association between fatigue and quality of life in terminally ill patients with cancer admitted to a palliative care unit

**DOI:** 10.1017/S1478951526102740

**Published:** 2026-06-09

**Authors:** Mizuki Matsuda, Yu Koyama, Nao Seki, Sayuri Sakai

**Affiliations:** 1 Graduate School of Health Sciences, Department of Nursing, Doctoral Program, Niigata University, Niigata, Japan; 2 Graduate School of Health Sciences, Niigata University, Niigata, Japan

**Keywords:** Cancer-related fatigue, terminal cancer, assessment, quality of life, physical fatigue

## Abstract

**Background:**

Cancer-related fatigue (CRF) is a distressing symptom in terminally ill patients with cancer. While many studies have been conducted on the relationship between fatigue and quality of life (QOL) among patients undergoing treatment, only a few have been conducted to measure fatigue from multiple perspectives or clarify its impact on patients in advanced stages or those receiving palliative care.

**Objectives:**

To examine the impact of fatigue on QOL in terminally ill patients with cancer, using a questionnaire that measures CRF from 3 perspectives.

**Methods:**

CRF and QOL were measured using the Cancer Fatigue Scale (CFS) and the European Organization for Research and Treatment of Cancer Quality of Life Questionnaire Core 15 Palliative (EORTCQLQ-C15-PAL), respectively, and the correlation between them was evaluated. Web-based questionnaires were completed by patients receiving hospice and palliative care.

**Significance of results:**

Twenty-nine participants provided valid responses. Median CFS and global health status/QOL scores were as follows: total fatigue 25.0, physical fatigue 11.0, affective fatigue 9.0, cognitive fatigue 5.0, and global health status/QOL 50.0. QOL showed significant correlations with total fatigue (*rs* = −0.44, *p =* 0.017, 95% CI: −0.70, −0.09) and physical fatigue (*rs* = −0.38, *p* = 0.038, 95% CI: −0.66, −0.02), but none with affective fatigue (*rs* = −0.02, *p* = 0.917, 95% CI: −0.38, 0.35) and cognitive fatigue (*rs* = −0.33, *p* = 0.074, 95% CI: −0.63, 0.03).

**Conclusions:**

Of the 3 aspects of fatigue, physical fatigue may be most closely related to QOL; however, its accurate assessment may depend on the scale used. Therefore, it is necessary to select an appropriate scale for patients with terminal cancer.

## Introduction

Cancer is a serious health issue affecting millions of people annually. In 2022, approximately 20 million new cases of cancer were reported, alongside 9.7 million related deaths (Bray et al. 2024). The quality of life (QOL) of patients with cancer is influenced by various factors, among which cancer-related fatigue (CRF) is particularly critical. The National Comprehensive Cancer Network (NCCN) defines CRF as “a distressing, persistent, subjective sense of physical, emotional, and/or cognitive tiredness or exhaustion related to cancer and/or cancer treatment that is not proportional to recent activity and interferes with usual functioning” (NCCN 2025).

Fatigue significantly impairs both physical and psychological functioning, limiting patients’ ability to carry out daily activities and consequently resulting in decreased treatment satisfaction and QOL (Lundh Hagelin et al. 2006; Hoekstra et al. 2007; Raaf et al. 2012). In particular, individuals in the terminal phase of cancer experience numerous physical and psychological symptoms, with fatigue being more distressing than other symptoms. In addition, CRF significantly impairs QOL, comparable to the impact of pain or depression (Lundh Hagelin et al. 2006; Hoekstra et al. 2007).

While many studies have been conducted on the relationship between fatigue and QOL among patients in the treatment phase (Stasi et al. 2003; Muthanna et al. 2023), there is a lack of studies on patients in advanced stages of the disease or those receiving palliative care. For patients in the advanced stage of the disease, participation in research studies is likely to impose significant physical and psychological burdens. The majority of the previous studies on this topic examined retrospective and outpatient data. The impact of fatigue on the QOL of patients with cancer at the end of life, who are expected to improve their QOL to live their final days as peacefully and independently as possible, has not yet been fully elucidated.

Several studies have shown that fatigue is negatively correlated with QOL in patients with cancer. However, some of these studies involved assessment using 1-dimensional scales such as the Visual Analogue Scale, which measures fatigue from a single aspect ( Strömgren et al. 2002; Iwase et al. 2015; Ahlam et al. 2019). The relationship between QOL and multiple aspects of fatigue has not been clarified. Fatigue is underestimated, and health-care providers do not fully understand the extent of its impact (Vogelzang et al. 1997; Williams et al. 2016). To improve QOL in patients with cancer, it is necessary to raise awareness of fatigue, understand its full impact on QOL, and provide care to alleviate it. However, only a few studies have been conducted to examine the impact of fatigue on QOL in terminally ill patients with cancer.

The aim of the present study was to determine the impact of fatigue on QOL in terminally ill patients with cancer, using a questionnaire survey that measured fatigue from 3 perspectives.

## Materials and methods

### Study design and population

This cross-sectional study was conducted between March 2025 and June 2025. To comprehensively assess the current state of palliative care units in Japan, a nationwide survey targeting all palliative care units was conducted. This approach aimed to achieve broad sample representativeness by avoiding facility-specific bias that often occurs in localized multicenter studies. A web-based survey using Google Forms was distributed to 2 participants (1 male and 1 female) from each of the 384 hospice and palliative care facilities registered as regular members of the Japan Hospice Palliative Care Association. Compared to men, women experience fatigue at a higher rate and with greater severity (Yuxia et al. 2020; Kang et al. 2023). To minimize the potential bias due to sex imbalance, 1 male and 1 female respondent were selected from each facility. Requests for research cooperation were sent to oncologists or administrators in palliative care units. The questionnaires were distributed to and collected from participants through participating facilities without direct involvement from the researchers. Consequently, the number of facilities that agreed to participate and actually distributed the questionnaires was not recorded. In addition, the number of patients who were approached, and who consented and completed the questionnaires were not recorded. Terminally ill patients with cancer admitted to the palliative care unit were eligible if they met the following inclusion criteria: 18 years or older, prognosis estimated in months by the oncologist, received hospice care, ability to complete the online questionnaire, and absence of severe mental or cognitive disorders. A number field was included in the web survey to detect duplicate responses.

The sample size required for detecting a correlation coefficient was calculated using G*Power 3.1. Assuming a 2-tailed alpha error of 0.05, test power (1-β) of 0.8, and expected effect size of 0.50, the calculated minimum required sample size was 26.

### Data collection

Patient information included age, sex, performance status (PS), and emotional distress. Emotional distress on a scale of 0–10, choosing 1 number to indicate how difficult you are feeling right now. A higher number indicates greater distress. The following assessment tools were used: the Cancer Fatigue Scale (CFS) and the Japanese Version of the European Organization for Research and Treatment of Cancer Quality of Life Questionnaire Core 15 Palliative (EORTCQLQ-C15-PAL) (Okuyama et al. 2000; Miyashita et al. 2015).

The CFS is a 15-item self-rating scale developed by Okuyama et al. to assess fatigue in patients with cancer. It comprises 3 subscales: physical, affective, and cognitive. The reliability and validity of this scale have been tested. Patients were asked to circle a number describing their current state on a scale of 1 (not at all) to 5 (very much). Total fatigue score is calculated as the sum of all subscale scores (maximum score: 60), with higher scores indicating more severe fatigue. A cut-off value of 19 points was used, and individuals with score ≥ 19 were considered to have severe fatigue, indicating interference with activities of daily living (Okuyama et al. 2001). The validity and reliability of this scale were further confirmed in a developmental study (Okuyama et al. 2000), in which only patients participated.

The EORTCQLQ-C15-PAL was developed by the European Organization for Research and Treatment of Cancer (EORTC) as an abbreviated version of the EORTCQLQ-C30 (Miyazaki et al. 2012). It is recommended for patients with advanced, incurable, and symptomatic cancer who have a median life expectancy of a few months. The questionnaire consists of 15 items covering 2 functional subscales (physical and emotional), global health status/QOL, and 7 symptom subscales (fatigue, pain, nausea/vomiting, dyspnea, appetite loss, sleep disturbance, and constipation). Each item is scored on a 4-point scale (1 = not at all; 2 = a little; 3 = quite a bit; 4 = very much), with higher scores indicating worse QOL. The global health item is scored separately on a 7-point scale (1 = very poor to 7 = excellent). This scale was chosen because it has been used in many studies examining patients admitted to palliative care units and it contains few questions. Permission to use the scale was obtained from the EORTC Quality of Life Group. This scale has been translated and used in various languages, and the reliability and validity of the Japanese version have been verified (Ghoshal et al. 2016).

### Statistical analysis

All data were analyzed using EZR (Easy R) version 1.68 (Saitama Medical Center, Jichi Medical University, Saitama, Japan). Statistical significance was set at *p* < 0.05. Descriptive statistics (frequency distribution, percentage, and mean [standard deviation, SD]) were used to summarize the demographic characteristics.

Spearman’s rank correlation coefficient was used to evaluate the association of CFS scores (subscale and total) with functional and symptomatic measures, including pain and global health status/QOL scores. The 95% confidence interval for the correlation coefficient was calculated and reported using an approximation based on Fisher’s *z*-transformation.

The dependent variable was overall global health status/QOL, and linear regression models were used to determine the impact of the 3 dimensions of fatigue on overall QOL.

Simple regression analysis was performed to examine the effect of CRF on QOL. The dependent variable was global health status/QOL in all models, and the independent variables were total fatigue and the 3 CFS subscale scores. Moreover, we conducted a regression analysis to examine the relationship between fatigue and QOL, after adjusting for PS and pain. Simple regression analysis was performed using the least squares method (lm function) to determine the regression coefficient (β), 95% confidence interval, *p*-value, and coefficient of determination (*R*^2^).

### Ethical considerations

This study was approved by the Ethics Committee of our University (Approval No. 2024-0231). Participants were informed about the study’s purpose, methods, and data protection measures, and the voluntary nature of their participation. Consent to participate was obtained through their response to the survey.

## Results

### Patient characteristics

Among cancer patients admitted to the palliative care unit, responses were obtained from 30 individuals who consented to participate in the study. One of whom was excluded owing to missing questionnaire item, leaving 29 patients for analysis (Table 1).

**Table 1.** Characteristics of participants Table 1 long description.

Characteristics	(*N* = 29)	(%)
Sex		
Men	12	41.4
Women	17	58.6
Age (years)		
50–60	6	20.7
61–70	10	34.5
71–80	11	37.9
81–90	2	6.9
ECOG performance status		
1	5	17.2
2	5	17.2
3	18	62.1
4	1	3.5
		Mean ± SD
Emotional distress		4.7 ± 2.9
Pain (EORTC QLQ-C30)		46.0 ± 33.5
Dyspnea (EORTC QLQ-C30)		42.5 ± 36.6

Emotional distress on a scale of 0 to 10, choosing 1 number to indicate how difficult you are feeling right now.

### Cancer fatigue scale and EORTC QLQ-C15-PAL scores

The median scores for the CFS subscales were as follows: total fatigue 25.0, physical fatigue (subscale) 11.0, affective fatigue 9.0, and cognitive fatigue 5.0 (Table 2). A cut-off score of 19 points was used to examine the incidence and severity of CRF. Five patients scored below 19 points, while 24 patients scored 19 points or higher. Results showed that 24 patients (82.8%) scored above this point, indicating a high prevalence of CFS in this sample.

**Table 2.** Cancer fatigue scale and EORTC QLQ-C15-PAL score Table 2 long description.

Cancer-related fatigue scales	Median	Q1 (25%)	Q3 (75%)	Min	Max
Total fatigue	25.0	19.0	32.0	11.0	46.0
Physical fatigue	11.0	7.0	15.0	4.0	25.0
Affective fatigue	9.0	7.0	10.0	3.0	15.0
Cognitive fatigue	5.0	3.0	9.0	0.0	14.0
EORTCQLQ-C15-PAL					
Global quality of life	50.0	33.3	83.3	0.0	100.0
Functional subscales					
Physical function	33.3	11.1	66.7	0.0	88.9
Emotional function	66.7	33.3	83.3	0.0	100.0
Symptom subscales					
Fatigue	66.7	33.3	66.7	16.7	100.0
Insomnia	33.3	33.3	66.7	0.0	100.0
Pain	33.3	16.7	83.3	0.0	100.0
Appetite loss	33.3	0.0	66.7	0.0	100.0
Dyspnea	33.3	0.0	66.7	0.0	100.0
Constipation	33.3	0.0	66.7	0.0	100.0
Nausea and vomiting	0.0	0.0	33.3	0.0	100.0

Table 2 shows the median scores and interquartile ranges (IQR, 25th–75th percentile) for each item. The median represents the central tendency of the data, while the IQR indicates the spread of the middle 50% of scores. Minimum and maximum values are also presented to describe the full range of observed scores.

For the EORTC QLQ-C15-PAL, the median global health status/QOL score was 50.0. The highest median symptom score was for fatigue 66.7 (Table 2). Symptom analysis showed that all patients reported some degree of fatigue.

### Relationship between quality of life (EORTC QLQ-C15-PAL) and fatigue measured by CFS

**Figure 1.**
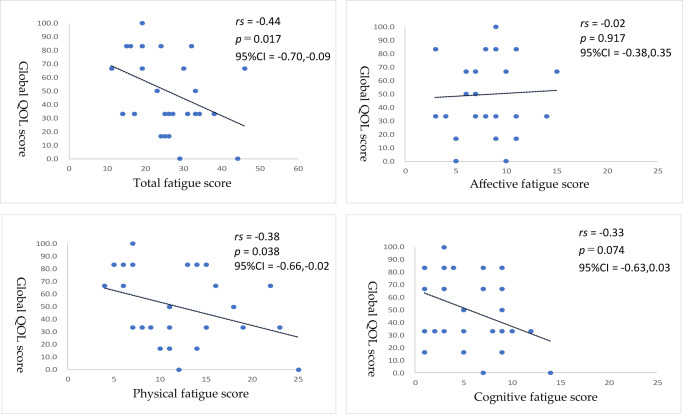
Distribution and association between Global QOL score and CFS score. Figure 1 long description.

Correlation analysis showed that global health QOL was significantly associated with total fatigue (*rs* = −0.44, *p* = 0.017, 95% CI: −0.70, −0.09). Physical fatigue was also significantly correlated with global health QOL (*rs* = −0.38, *p* = 0.038, 95% CI: −0.66, −0.02). In contrast, neither affective fatigue (*rs* = −0.02, *p* = 0.917, 95% CI: −0.38, 0.35) nor cognitive fatigue (*rs* = −0.33, *p* = 0.074, 95% CI: −0.63, 0.03) showed a statistically significant association with global health QOL (Figure 1).

* Spearman’s correlation coefficients (rs) were calculated for Global QOL score and each fatigue subscale score (*p* < 0.05).CFS, the Cancer Fatigue Scale; EORTCQLQ-C15-PAL; European Organization for Research and Treatment of Cancer Quality of Life Questionnaire Core 15 Palliative.

These results indicated a negative correlation between total fatigue and physical fatigue. On the other hand, both emotional fatigue and cognitive fatigue no statistically significant association with QOL was observed in this sample. Regression analysis showed that CRF was negatively associated with QOL in terminally ill patients with cancer. Specifically, total fatigue had a negative coefficient (*β* = − 1.37, 95% CI: −2.55, −0.19, *p* = 0.024), suggesting that higher overall fatigue levels were associated with lower QOL. The model *R*^2^ was 0.172, and the fit was somewhat low. For the 3 CFS subscales, physical fatigue (*β* = − 1.99, 95% CI: −3.39, −0.42, *p* = 0.032), affective fatigue (*β* = 0.21, 95% CI: −3.24, 3.51, *p* = 0.911), and cognitive fatigue (β = −2.98, 95% CI: −5.27, −0.12, *p* = 0.041) were observed. When each CFS subscale was analyzed independently, physical and cognitive fatigue showed significant negative correlations with QOL, with negative coefficients and *p*-values < 5%. To examine whether the observed associations were independent of potential confounders, additional regression analyses were performed adjusting for PS and pain. After adjusting for PS, CRF remained significantly associated with QOL (*β* = −1.38, 95% CI: −2.59, −0.16, *p* = 0.028). Even after adjusting for pain, CRF remained significantly associated with QOL (*β* = −1.34, 95% CI: −2.53, −0.16, *p* = 0.028).

### Relationship between quality of life (EORTC QLQ-C15-PAL) and fatigue measured by EORTC QLQ-C15-PAL

No significant correlation between QLQ-C15-PAL subscale about fatigue and global health QOL (*rs* = −0.33, *p* = 0.079).

### Relationship between quality of life and other symptoms

The correlation between each QLQ-C15-PAL subscale and global health QOL was positive for emotional functioning (*rs* = 0.47, *p* = 0.009) and negative for nausea/vomiting (*rs* = −0.60, *p* < 0.001), insomnia (*rs* = −0.43, *p* = 0.028), and constipation (*rs* = −0.40, *p* = 0.029) (Table 3).

**Table 3.** The correlation between each EORTC QLQ-C15-PAL subscale and the global health of QoL Table 3 long description.

	*rs*	*p*
Functional subscales		
Physical function	0.10	0.599
Emotional function	0.47	0.009
Symptom subscales		
Fatigue	−0.33	0.079
Insomnia	−0.43	0.028
Pain	−0.20	0.289
Appetite loss	−0.26	0.166
Dyspnea	−0.20	0.298
Constipation	−0.40	0.029
Nausea and vomiting	−0.60	<0.001

Calculate Spearman’s coefficient between QOL on the EORTC QLQ-C15-PAL subscale and physical function, emotional function, and symptoms (*p* < 0.05).

## Discussion

The aim of the present study was to examine the association between fatigue and QOL in terminally ill patients with cancer, using a questionnaire that measured fatigue from 3 perspectives. The results showed that total and physical fatigue tended to be negatively associated with QOL.

CRF is one of the most distressing and prevalent symptoms in terminally ill patients with cancer (Iwase et al. 2015; Ahlam et al. 2019), and it is a complex construct comprising several components. The results of this study suggest that CRF is associated with lower QOL and consistent with those of previous studies (Gupta et al. 2007; Karthikeyan et al. 2012; Iwase et al. 2015; Charalambous and Kouta 2016; Ghoshal et al. 2016; Agarwal et al. 2020). Among the components of fatigue, physical fatigue (feeling tired, exhausted, reluctant, and fed up) was associated with QOL and showed a significant negative regression coefficient for QOL in both correlation analysis and linear regression analysis among terminally ill patients with cancer. Physical limitations restrict individuals from continuing normal lifestyles and affect their ability to perform daily activities. These limitations lead to distress through loss of independence and increased dependence, and are therefore considered to be related to QOL. The model explained 17% of the variance in the global QOL (*R*^2^ = 0.17), which is acceptable given the multifactorial nature of QOL in palliative cancer care. Despite the modest explanatory power, explained approximately 17% of the variance in QOL, indicating that fatigue may contribute to variations in perceived QOL. Given that QOL is influenced by a range of factors – including pain, dyspnea, appetite loss, and social circumstances – this level of explained variance is not unexpected. Although the association between fatigue and QOL remained significant after the adjustment for PS and pain, the findings should be interpreted with caution due to the small sample size. Although no correlation was observed between cognitive fatigue and QOL, regression analysis suggested the possibility of a decline in QOL. While regression analysis directly uses the magnitude of observed values to test the slope of a linear relationship, Spearman’s rank correlation coefficient converts values into ranks to test for the presence of a monotonic relationship. Therefore, the relationship is likely primarily linear, information was lost through rank transformation, the data contain ties or measurement errors, or the relationship is influenced by the distribution or outliers. No outliers were identified in this study. Therefore, while the results were significant in the regression analysis, this is considered due to differences in the characteristics of the methods rather than anomalies in the data.

Regarding affective fatigue, neither correlation analysis nor regression analysis revealed any association with QOL. This result is considered reasonable given the factors associated with QOL in terminally ill patients receiving palliative care and the characteristics of the assessment tool used. First, the overall QOL item of the QLQ-C15-PAL is a comprehensive indicator strongly influenced by physical aspects such as physical symptoms, functional status, and general health, and is not structured to directly and specifically reflect psychological aspects. Therefore, the impact of the “affective fatigue” experienced by patients on QOL levels may tend to be relatively small. In contrast, physical fatigue is more directly linked to limitations in activities of daily living and reduced activity levels, making it more likely to have an immediate impact on the patient’s own QOL. Second, for patients in the terminal phase, physical symptoms such as pain, dyspnea, physical fatigue, and insomnia often constitute the primary determinants of QOL (Stone et al. 1999; Annette et al. 2002; Knobel et al. 2003; Peters and Sellick 2006). Consequently, even if a potential association between affective fatigue and QOL exists, it may be obscured by the strong association with physical symptoms and thus difficult to detect through simple correlation analysis. Furthermore, psychological variables often exhibit small effect sizes, and the sample size of this study, although meeting the required threshold, may not have provided adequate power to detect such subtle associations.

Therefore, the lack of a significant correlation between affective fatigue and QOL can be interpreted as a valid and understandable result, influenced by multiple factors including the characteristics of the measurement scales, the symptom structure of palliative care patients. On the other hand, physical fatigue was shown to have a moderate association with QOL, reaffirming the importance of fatigue management in palliative care. Future studies should consider using scales that allow for a more detailed assessment of the psychological domain of QOL, conducting multivariate analyses adjusted for physical symptoms, and expanding the sample size.

An interesting finding of this study was the difference in the relationship between QOL and fatigue scores measured by the CFS and EORTC QLQ-C15-PAL. Total fatigue measured using the CFS had a significant impact on QOL, with a significant negative correlation. In contrast, fatigue measured using the EORTC QLQ-C15-PAL showed no significant correlation. This may be because the CFS is a multidimensional scale that evaluates fatigue using 15 questions, whereas the EORTC QLQ-C15-PAL assesses fatigue using only 2 questions: “Do you feel your physical strength has decreased?” and “Were you tired?” This suggests that the EORTC QLQ-C15-PAL may not adequately capture fatigue in terminally ill patients with cancer. Although this shorter version was selected to reduce patient burden; however, the longer version (EORTC QLQ-C30) has been reported to have a ceiling effect in palliative care patients and is therefore not recommended as a single measure in this population (Knobel et al. 2003). Similar concerns may extend to the EORTC QLQ-C15-PAL, despite having fewer questions.

Numerous scales are available for the clinical evaluation of fatigue associated with cancer. Single-item unidimensional scales are commonly used for determining fatigue. Multidimensional scales, which consider the multiple dimensions of fatigue, provide a more comprehensive assessment; however, but they have limited scope in their usage (Minton and Stone 2009). Physical fatigue is most closely associated with QOL and is often observable by health-care providers. Moreover, more time is required to collect data because of the large number of items (D’Silva et al. 2022).

Patients in the palliative phase experience several unique and distressing symptoms compared with survivors or patients undergoing active treatment. In the terminal stage, self-assessment of symptoms becomes difficult; therefore, health-care providers must understand that fatigue is one of the symptoms that may be underestimated and inadequately managed, and that it is necessary to assess and manage fatigue from multiple perspectives.

Recent advancements in medical philosophy have increasingly focused on mitigating the suffering of terminally ill patients and guiding them towards a dignified acceptance of death. Our study suggests that fatigue may be a factor contributing factor to reduced QOL in patients, indicating the need for care addressing fatigue. However, patients with cancer are not being informed by health-care providers that fatigue is an untreatable symptom and must be endured as an inevitable part of cancer and its treatment (Agarwal et al. 2020). Previous studies have found barriers to reporting fatigue among patients with cancer (Passik et al. 2002; Milzer et al. 2024). Health-care providers must bear in mind that patients find it difficult to report fatigue. Health-care providers need to build a trusting relationship with patients from the onset and try to create an environment in which patients can report various symptoms, including fatigue.

### Limitations

This study retains several limitations. Although we conducted regression analyses adjusting for PS and pain, other potential confounding factors (e.g., dyspnea and psychological distress) were not included in the analysis. Therefore, the observed associations might be subject to the influence of residual confounding.

In a survey conducted in Japan during fiscal year 2019, the average length of stay was 28.5 days, and 63% of facilities had an average length of stay of less than 30 days. The average mortality rate was high at 78.7%, suggesting that most patients with cancer were at the very end of life (Japan Hospice Palliative Care Foundation 2025). Under these circumstances, many patients had difficulty completing questionnaires, resulting in challenges in achieving a large sample size. Thus, although this study met the pre-calculated required sample size and the analyses were considered statistically valid, the findings should be interpreted with caution due to the restricted conditions under which the data were collected.

The representativeness of the sample is also limited. To minimize the potential bias due to sex imbalance (Yuxia et al. 2020; Kang et al. 2023), 1 male and 1 female respondent were selected from each facility. Although this approach reduced the risk of sex-related bias, it may have introduced selection bias by artificially fixing the sex ratio at 1:1, regardless of the actual distribution in the patient population. Therefore, even statistically significant results cannot be directly generalized to the broader population.

Furthermore, since this study comprised only patients who were able to complete the online questionnaire, the participants might have represented a subgroup of terminally ill patients who were in relatively good physical condition. As a result, patients experiencing severe fatigue might have been underestimated, thereby potentially having led to non-response bias and underestimating the association between fatigue and QoL.

The purpose of this study was to clarify the relationship between QOL and fatigue in patients with cancer; therefore, differences by cancer type were not a primary focus. As many of the participants were receiving terminal care, it was necessary to minimize response burden. Moreover, given the limited sample size, analysis by cancer type was not feasible, and therefore, information on cancer type was not collected. Given that different cancer types and treatment modalities may affect patient with CFS, future research should consider these variations.

Finally, this study analyzed responses from 29 patients and is considered a preliminary investigation. Nevertheless, given the scarcity of research involving patients admitted to PCUs and considering the high mortality rate and restricted recruitment environment, the findings provide valuable insight. Future multicenter studies with larger and more diverse samples are needed to enhance the generalizability of the results.

## Conclusions

Our findings revealed that total and physical fatigue were negatively correlated with QOL, with physical fatigue being the most associated; however, its accurate assessment may depend on the scale used. Therefore, it is necessary to select a scale that is appropriate for the specific patient population. Fatigue is a multifaceted symptom that is easily overlooked, making it essential for health-care providers to understand its impact on QOL.

Accurate understanding and appropriate assessment of fatigue are crucial for developing individualized care plans and implementing targeted interventions, thereby preventing fatigue-related declines in QoL. Health-care professionals should understand and evaluate the impact of fatigue on QOL to ensure effective management.

## Table 1 Long description

Participant characteristics are summarized for 29 people, reporting counts and percentages for sex, age groups, and ECOG performance status, plus mean and standard deviation for symptom scores. Women made up 17 participants (58.6%) and men 12 (41.4%). Ages were concentrated between 61 and 80 years: 10 participants were 61 to 70 (34.5%) and 11 were 71 to 80 (37.9%); fewer were 50 to 60 (20.7%) or 81 to 90 (6.9%). ECOG performance status was mostly 3, with 18 participants (62.1%), while statuses 1 and 2 each had 5 participants (17.2%), and status 4 had 1 participant (3.5%). Mean emotional distress was 4.7 with a standard deviation of 2.9 on a 0 to 10 scale. Mean symptom scores were 46.0 (standard deviation 33.5) for pain and 42.5 (standard deviation 36.6) for dyspnea on the EORTC QLQ-C30. Percentages may not sum to exactly 100 due to rounding.

Navigate back to Table 1.

## Table 2 Long description

The table summarizes cancer-related fatigue scores (total, physical, affective, cognitive) and EORTC QLQ-C15-PAL quality-of-life, function, and symptom scores using medians, interquartile ranges, and minimum to maximum values. Total fatigue has a median of 25.0 with a middle spread from 19.0 to 32.0, ranging from 11.0 to 46.0. Physical fatigue is the largest fatigue component (median 11.0) compared with affective fatigue (median 9.0) and cognitive fatigue (median 5.0, including a minimum of 0.0). Global quality of life has a median of 50.0 with wide variability from 0.0 to 100.0. Among functional subscales, emotional function is higher (median 66.7) than physical function (median 33.3). Among symptom subscales, fatigue is highest (median 66.7), while nausea and vomiting is lowest (median 0.0) though it can reach 100.0. Several symptoms such as insomnia, pain, appetite loss, dyspnoea, and constipation have medians around 33.3 with broad ranges up to 100.0. Interpretation should note that medians describe typical scores and the wide ranges indicate substantial differences between individuals.

Navigate back to Table 2.

## Figure 1 Long description

The image A showing a scatter plot with the vertical axis labeled Global QOL score and tick labels from 0.0 to 100.0. The horizontal axis is labeled Total fatigue score with tick labels 0, 10, 20, 30, 40, 50, 60. A fitted line slopes downward from left to right. Text on the plot reads: rs equals minus 0.44; p equals 0.017; 95 percent CI equals minus 0.70, minus 0.09. The image B showing a scatter plot with the vertical axis labeled Global QOL score and tick labels from 0.0 to 100.0. The horizontal axis is labeled Affective fatigue score with tick labels 0, 5, 10, 15, 20, 25. A fitted line slopes slightly upward from left to right. Text on the plot reads: rs equals minus 0.02; p equals 0.917; 95 percent CI equals minus 0.38, 0.35. The image C showing a scatter plot with the vertical axis labeled Global QOL score and tick labels from 0.0 to 100.0. The horizontal axis is labeled Physical fatigue score with tick labels 0, 5, 10, 15, 20, 25. A fitted line slopes downward from left to right. Text on the plot reads: rs equals minus 0.38; p equals 0.038; 95 percent CI equals minus 0.66, minus 0.02. The image D showing a scatter plot with the vertical axis labeled Global QOL score and tick labels from 0.0 to 100.0. The horizontal axis is labeled Cognitive fatigue score with tick labels 0, 5, 10, 15, 20, 25. A fitted line slopes downward from left to right. Text on the plot reads: rs equals minus 0.33; p equals 0.074; 95 percent CI equals minus 0.63, 0.03.

Navigate back to Figure 1.

## Table 3 Long description

The table reports Spearman correlations between global health quality of life and EORTC QLQ-C15-PAL functional and symptom subscales, with p values for each association. Emotional function shows a moderate positive correlation of 0.47 with a p value of 0.009, while physical function shows a weak positive correlation of 0.10 with a p value of 0.599. Among symptoms, nausea and vomiting has the strongest association, a moderate negative correlation of minus 0.60 with a p value below 0.001. Insomnia and constipation also show moderate negative correlations, minus 0.43 and minus 0.40, with p values of 0.028 and 0.029. Fatigue, pain, appetite loss, dyspnoea, and constipation are all negative, but fatigue, pain, appetite loss, and dyspnoea are not statistically significant based on their p values of 0.079, 0.289, 0.166, and 0.298. Overall, higher symptom burden tends to align with lower global health, while better emotional function aligns with higher global health; correlations do not establish causation.

Navigate back to Table 3.
